# Psoriasis Increased the Risk of Adverse Cardiovascular Outcomes: A New Systematic Review and Meta-Analysis of Cohort Study

**DOI:** 10.3389/fcvm.2022.829709

**Published:** 2022-03-25

**Authors:** Lu Liu, Saijin Cui, Meitong Liu, Xiangran Huo, Guoqiang Zhang, Na Wang

**Affiliations:** ^1^Cancer Institute, The Fourth Hospital of Hebei Medical University, Shijiazhuang, China; ^2^Department of Dermatology, The First Hospital of Hebei Medical University, Shijiazhuang, China; ^3^Candidate Branch of National Clinical Research Center for Skin Diseases, Beijing, China

**Keywords:** cardiovascular disease, cohort study, meta-analysis, systematic review, psoriasis

## Abstract

**Background:**

Several studies have investigated the relationship between psoriasis and adverse cardiovascular outcomes. Previous meta-analyses have shown psoriasis to be a risk factor for adverse cardiovascular outcomes. However, the relationship has become uncertain with the emergence of many new studies.

**Objective:**

This study aimed to conduct an updated meta-analysis on cohort studies about the relationship between psoriasis and adverse cardiovascular outcomes.

**Methods:**

Electronic databases (accessed till January 2022) were searched systematically for cohort studies assessing the cardiovascular risk in psoriasis patients. This was a meta-analysis using a random-effect model; pooled analyses of several cardiovascular outcomes were also conducted.

**Results:**

A total of 31 [hazard ratio (HR), 23; rate ratio (RR), 8] studies involving 665,009 patients with psoriasis and 17,902,757 non-psoriatic control subjects were included for the meta analysis. The pooled analyses according to each cardiovascular outcome revealed that pooled RR of patients for developing myocardial infarction, stroke, cardiovascular death, ischemic heart disease, thromboembolism and arrhythmia were 1.17 (95% confidence interval [CI], 1.11–1.24), 1.19 (95% CI, 1.11–1.27), 1.46 (95% CI, 1.26–1.69), 1.17 (95% CI, 1.02–1.34), 1.36 (95% CI, 1.20–1.55) and 1.35 (95% CI, 1.30–1.40), respectively. Meanwhile, the pooled RR of patients with mild and severe psoriasis for developing adverse cardiovascular outcomes were 1.18 (95% CI, 1.13–1.24) and 1.41 (95% CI, 1.31–1.52), respectively.

**Conclusion:**

The pooled analyses revealed that psoriasis is associated with all adverse cardiovascular outcomes of interest, especially in severe patients. Psoriasis remains an independent risk factor for adverse cardiovascular outcomes, which needs more attention from clinicians.

## Introduction

Psoriasis is a chronic inflammatory disease (1) which affects sixty million adults and children worldwide (2). It is mainly characterized by dry, erythematous, round or scaly patches of the skin. The disease affects the daily activities and sleeps of patients and significantly reduces their quality of life (3). Studies have shown that psoriasis shares the same risk factors with cardiovascular disease (CVD), such as hypertension, obesity, smoking, and diabetes mellitus (4). Moreover, psoriasis also shares a similar immune-inflammatory mechanism with atherosclerosis, one of the CVDs, which all involve T-helper cell type 1 and type 17 activation and decreased T-regulatory cell function (4, 5). Therefore, psoriasis might be an independent risk factor for the development of adverse cardiovascular outcomes.

Generally speaking, CVD is a group of disorders of the heart and blood vessels, including coronary artery disease, cerebrovascular disease, peripheral artery disease, and aortic atherosclerosis. Unexpected sudden events caused by CVD such as myocardial infarction, stroke, cardiovascular death etc. are also called cardiovascular events (CVE) in clinic practice. CVD is a major public health concern that affecting millions of people worldwide. It was the leading cause of death in the twenty-first century, accounting for about a third of all global deaths (6). Obesity and hypertension are known and established risk factors for CVD (7–10); however, due to the complex etiology of CVD, other related risk factors still need to be further recognized and confirmed. So far, the correlation between psoriasis and CVD was investigated by several studies, but the results were inconsistent (11–13). Although two meta-analyses summarized the relationship between psoriasis and cardiovascular outcomes, they included more case–control studies than cohort studies (14, 15). An updated meta-analysis was needed to confirm this relationship with the emergence of many new clinical studies. Hence, we intended to conduct a meta-analysis of the original cohort studies published before January 2022, which represents the highest level of evidence of the relationship between psoriasis and adverse cardiovascular outcomes among all observational studies.

## Materials and Methods

In our study, the exposure factor was psoriasis, and the outcome was adverse cardiovascular outcomes. Therefore, we used psoriasis patients as the exposure group and non-psoriatic subjects as the control group. We used the Preferred Reporting Items for Systematic Reviews and Meta-Analyses (PRISMA) as a guideline for our study and mapped the PRISMA flow diagram.

### Data Source and Search

The searches were performed in collaboration with an informatics specialist in four electronic databases (MEDLINE, EMBASE, SCI–Web of science and the Cochrane Library) for all relevant studies on psoriasis and adverse cardiovascular outcome published before January 2022. The records were screened by two reviewers independently using Endnote 9.1.

Our retrieval strategy consisted of three modules, each of which was required to contain at least one of the following terms: “psoriasis” OR “Psoriases” OR “Pustulosis of Palms and Soles” OR “Pustulosis Palmaris et Plantaris” OR “Palmoplantaris Pustulosis” OR “Pustular Psoriasis of Palms and Soles”; “cardiovascular diseases” OR “Cardiovascular Disease” OR “Disease, Cardiovascular” OR “Diseases, Cardiovascular”; “risk” OR “mortality” OR “mortality” OR “cohort”. Supplementary Table 1 summarized the specific retrieval strategies and results used for each database.

### Inclusion/Exclusion Criteria

#### Inclusion Criteria

Cohort studies about the relationship between psoriasis and cardiovascular outcome in adults (≥ 18 years old); psoriasis patients that were diagnosed in regular hospitals or had records in other medical and health institutions; the outcome indicator of the study was hazard ratio (HR) or rate ratio (RR). It has been shown that the practical meaning of HR and RR are very similar, so we converted HR directly to RR for merging and the final effect size is represented by RR (16).

#### Exclusion Criteria

Summaries, systematic reviews, conference abstract and studies with inappropriate control groups or lacking primary data were excluded. For research that lack original data, we contacted the author twice *via* email. Invalid email addresses or failure to receive a response would result in the exclusion of the research from the study. If there was more than one control group of psoriasis patients in the study, the most appropriate group was selected as the control group. If there were duplicates in the study, we selected only data that were more homogeneous with other studies.

### Definition of “Exposure” and “Outcome”

In our study, CVD included ischemic heart disease, aortic valve stenosis and atherosclerosis. CVE were defined as conditions such as cardiovascular death, angina pectoris, myocardial infarction, heart failure, arrhythmia, venous thromboembolism, aneurysm, stroke and transient ischemic attack. All of the above diseases served as the outcome events observed in this study.

Psoriasis was divided into three categories according to severity, namely, mild, moderate, and severe. “Mild” was defined as patients requiring only topical treatment, whereas “moderate” and “severe”, which were combined into one group, were patients requiring systemic treatment as well as those with psoriatic arthritis.

The diagnosis of all diseases must be based on the International Classification of Diseases (ICD) or other authoritative diagnostic basis.

### Data Extraction

The following information was obtained from each article: author, journal, year of publication, country of origin, selection and characteristics of psoriasis patients and non-psoriatic subjects, demographics, ethnicity, HR (or RR), and 95% confidence interval (CI). For subjects of different ethnicities, data were extracted separately and categorized as European and Asian. For articles in non-English languages, we translated them using Zhiyun translator (V7.0).

### Data Analysis

All analyses were conducted using the STATA statistics software V16.0 and Review manager V5.3 using random-effects model.

First, we tested for publication bias by funnel plots of Egger’s test and Begg’s test. If the *P*-value of the tests was less than 0.1, there was publication bias. In addition, the Egger’s test is usually considered more sensitive than the Begg’s test (17). We chose the result of Egger’s test if they were inconsistent.

The Newcastle-Ottawa scale (NOS, range 0--9)^1^ was used to assess the quality of each study. A score > 6 was regarded as high quality. Studies with a score ≤ 6 will be excluded.

Statistical heterogeneity was estimated with the *I*^2^ statistic (9), which was interpreted as follows: 25–75%, moderate heterogeneity; > 75%, substantial heterogeneity. Sensitivity analysis was also conducted to ensure the stability of the results. To effectively detect heterogeneity, we used a more conservative random-effects model (18).

In addition to comparisons of total number of subjects, we also classified the studies according to the ethnicity, severity of psoriasis, and cardiovascular outcomes for different analyses (if a particular group was included in only one or two published studies, it would be classified into the “Other” group for convenience). If a study contains data of two outcomes, it will be included in analyses for each outcome separately. For each group, we evaluated heterogeneity among studies using the χ^2^-based *Q*-test (19), and the heterogeneity was significant if *P* < 0.05.

To ensure that patients with psoriasis and no psoriasis were not counted several times, we selected data with the largest number of participants if a medical database was used by multiple studies in adjacent time periods and the number of psoriasis patients were similar.

### Ethics Approval Statement

Institutional review board approval was not required, given that the analysis was based on unidentified secondary data from previously published studies.

## Results

### Literature Search

**FIGURE 1:**
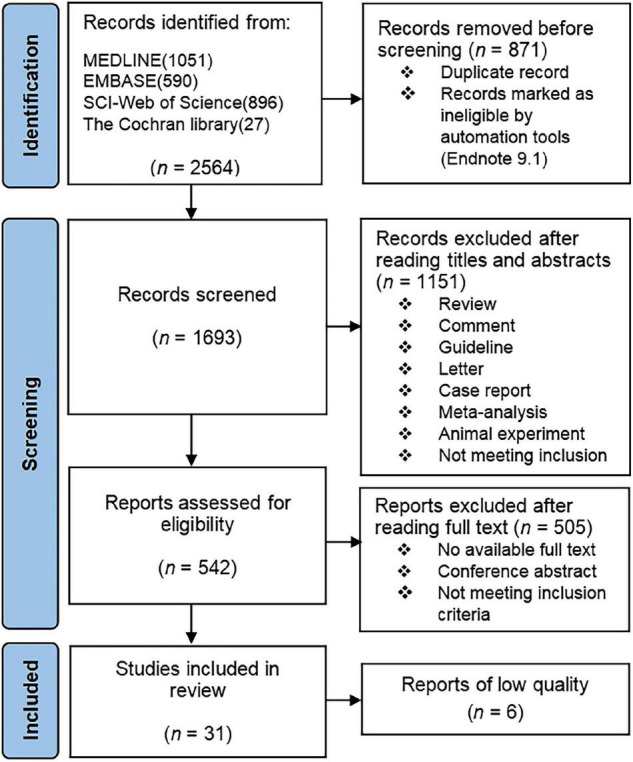
Flow diagram of the literature search and article evaluation process.

We found 2,564 eligible references in four databases; 2,533 references were excluded upon screening. The screening process is presented in Figure 1, and Table 1 summarized the characteristics of the 31 included studies. A total of 31 studies involving 665,009 psoriasis patients and 17,902,757 non-psoriatic control subjects were included in the meta-analysis (4, 12, 13, 20–47). The number of participants in studies with population duplication was not counted. Six low-quality articles were excluded (11, 48–52). Of the 31 studies, 9 were retrospective cohort studies, while the others were prospective. The mean follow-up time for the included studies was 7.1 years. Several studies had collected more than one observed outcome. Three relevant studies were excluded due to no access to full texts (53–55).

**TABLE 1:** Characteristics of included studies investigated the association between psoriasis and cardiovascular diseases/events risk.

References	Magazine	Country	Racial descent	Data resource	Psoriasis	No psoriasis	Severity	HR or RR (95% CI)	Outcome
Abuabara et al. (20)	Br J Dermatol	England	European	PB	3,603	14,330	2	1.57 (1.26–1.96)	Cardiovascular death
Ahlehoff et al. (42)	Eur Heart J	Denmark	European	PB	39,558	4,478,926	1	1.22 (1.14–1.30)	Atrial fibrillation
							2	1.53 (1.23–1.91)	Atrial fibrillation
							1	1.25 (1.17–1.34)	Stroke
							2	1.65 (1.33–2.05)	Stroke
Ahlehoff et al. (41)	J Intern Med	Denmark	European	PB	2,242	97,115	1	0.99 (0.87–1.11)	Thromboembolism
							2	1.27 (1.02–1.57)	Thromboembolism
							1	0.97 (0.80–1.16)	Stroke
							2	1.51 (1.02–2.05)	Stroke
Ahlehoff et al. (4)	J Intern Med	Denmark	European	PB	36,992	4,003,265	1	1.14 (1.06–1.22)	Cardiovascular death
							2	1.57 (1.27–1.94)	Cardiovascular death
							1	1.22 (1.12–1.33)	MI
							2	1.45 (1.10–1.90)	MI
							1	1.25 (1.16–1.33)	Stroke
							2	1.71 (1.39–2.11)	Stroke
Ahlehoff et al. (43)	PLoS One	Denmark	European	PB	38664	4,126,075	1	1.35 (1.21–1.49)	Venous thromboembolism
							2	2.06 (1.63–2.61)	Venous thromboembolism
Brauchli et al. (37)	Br J Dermatol	England	European	PB	36,702	36,702	0	1.07 (0.89–1.29)	MI
							0	0.92 (0.77–1.09)	Stroke
							0	0.98 (0.81–1.19)	Transient ischemic attack
Chiang et al. (21)	J Dermatol	China Taiwan	Asian	PB	2,783	13,910	0	1.27 (1.05–1.52)	Ischemic stroke
Chiu et al. (22)	J Am Acad Dermatol	China Taiwan	Asian	PB	40,637	162,548	2	1.34 (1.29–1.39)	Arrhythmia
Chiu et al. (23)	J Am Acad Dermatol	China Taiwan	Asian	PB	34,301	137,204	2	1.80 (1.25–2.61)	Aortic aneurysm
Chung et al. (24)	Thromb Haemost	China Taiwan	Asian	PB	8,945	8,945	0	2.02 (1.42–1.88)	Thromboembolism
Dregan et al. (44)	Circulation	England	European	PB	45,440	373,851	1	1.08 (0.98–1.18)	Stroke
							2	0.93 (0.64–1.36)	Stroke
							1	1.03 (0.97–1.11)	Coronary heart disease
							2	1.29 (1.01–1.64)	Coronary heart disease
Egeberg et al. (25)	Acta Derm Venereol	Denmark	European	PB	53,454	4,300,085	1	1.03 (0.96–1.11)	MI
							2	1.21 (1.05–1.39)	MI
Egeberg et al. (45)	J Am Acad Dermatol	Denmark	European	PB	30,278	2,692,097	1	1.27 (1.11–1.45)	MACE
							2	1.69 (1.20–2.37)	MACE
Gelfand et al. (39)	JAMA	England	European	PB	130,976	556,995	1	1.15 (1.10–1.20)	MI
							2	1.16 (1.11–1.21)	MI
Gelfand et al. (38)	J Invest Dermatol	England	European	PB	132,746	496,666	1	1.06 (1.00–1.10)	Stroke
							2	1.43 (1.10–1.90)	Stroke
Jung et al. (26)	J Dermatol	Korea	Asian	PB	5,788	1,727,832	1	0.96 (0.72–1.26)	MI
							2	2.24 (1.51–3.32)	MI
							1	1.09 (0.97–1.23)	Stroke
							2	1.23 (0.96–1.59)	Stroke
							1	1.25 (1.11–1.41)	Ischemic heart disease
							2	1.52 (1.21–1.92)	Ischemic heart disease
							1	1.32 (1.17–1.50)	Angina pectoris
							2	1.38 (1.06–1.79)	Angina pectoris
Kaye et al. (40)	Br J Dermatol	England	European	PB	44,164	219,784	0	1.21 (1.10–1.32)	MI
							0	1.20 (1.00–1.25)	Stroke
							0	1.20 (1.12–1.29)	Angina
							0	1.28 (1.20–1.48)	Atherosclerosis
Khalid et al. (27)	Eur J Heart Fail	Denmark	European	PB	66,389	5,376,842	1	1.22 (1.15–1.28)	Heart failure
							2	1.55 (1.36–1.76)	Heart failure
Khalid et al. (46)	Eur Heart J	Denmark	European	PB	70,665	5,036,959	1	1.22 (1.11–1.33)	Aortic valve stenosis
							2	1.61 (1.32–1.96)	Aortic valve stenosis
Khalid et al. (47)	Arterioscler Thromb Vasc Biol	Denmark	European	PB	70,989	5,404,544	1	1.20 (1.03–1.39)	Abdominal aortic aneurysm
							2	1.67 (1.21–2.32)	Abdominal aortic aneurysm
Leisner et al. (28)	JEADV	Denmark	European	PB	8,879	90,167	0	1.40 (1.09–1.80)	MI
Levesque et al. (29)	J Cutan Med Surg	Canada	European	PB	31,421	31,421	0	1.17 (1.04–1.31)	MI
							1	1.16 (0.94–1.42)	MI
							2	1.18 (1.05–1.33)	MI
Lin et al. (30)	J Am Acad Dermatol	China Taiwan	Asian	PB	4,752	23,760	0	2.10 (1.27–3.43)	MI
Lin et al. (31)	Int J Dermatol	America	European	PB	1,344	2,678	0	1.18 (0.80–1.74)	MI
							0	1.06 (0.85–1.33)	Heart failure
							0	1.04 (0.79–1.37)	Cardiovascular death
Mehta et al. (12)	Eur Heart J	England	European	PB	3,603	14,330	2	1.57 (1.26–1.96)	Cardiovascular death
Mehta et al. (32)	Am J Med	England	European	PB	3,603	14,330	2	1.53 (1.26–1.85)	MACE
Ogdie et al. (33)	Ann Rheum Dis	England	European	PB	138,424	81,573	1	1.09 (1.00–1.20)	Cardiovascular death
							2	1.54 (1.15–2.05)	Cardiovascular death
							1	1.08 (0.98–1.18)	MI
							2	1.26 (0.92–1.72)	MI
							1	1.08 (0.99–1.17)	Stroke
							2	1.45 (1.10–1.92)	Stroke
Parisi et al. (34)	J Invest Dermatol	England	European	PB	48,523	208,187	2	1.28 (0.96–1.69)	MACE
Rhee et al. (35)	Sci Rep	Korea	Asian	PB	13,385	739,459	1	1.10 (0.97–1.24)	Atrial fibrillation
							2	1.44 (1.14–1.82)	Atrial fibrillation
							1	1.04 (0.96–1.13)	Thromboembolic events
							2	1.26 (1.07–1.47)	Thromboembolic events
Wakkee et al. (36)	J Invest Dermatol	Holland	European	PB	15,820	27,577	0	0.94 (0.80–1.11)	MI
							0	1.05 (0.95–1.17)	Ischemic heart disease
Wu et al. (13)	J Dermatolog Treat	America	European	PB	14,014	70,070	1	1.28 (1.02–1.60)	MI
							2	1.31 (1.14–1.51)	MI

*In the “Severity” column, “1” represents patients with mild psoriasis, “2” represents patients with moderate to severe psoriasis, and “0” includes patients with all levels of severity of psoriasis. CI, confidence interval; PB, population-based; MACE, major adverse cardiovascular event; MI, myocardial infarction.*

### Quality Assessment

We conducted quality assessment of the 31 studies based on the NOS. The scores for these studies ranged from 7 to 9, and the mean of the quality scores was 7.94 (standard deviation ± 0.44). Table 2 summarized the specific items of NOS tool for each included study.

**TABLE 2:** The quality assessment of 31 included studies based on the Newcastle–Ottawa Scale (range 0–9).

References	Representativeness of the exposed cohort	Selection of the non-exposed cohort	Ascertainment of exposure	Demonstration that outcome of interest was not present at start of study	Comparability of cohorts on the basis of the design or analysis	Assessment of outcome	Was follow-up long enough for outcomes to occur	Adequacy of follow up of cohorts	Quality score
Abuabara et al. (20)	★	★	★	★	★	★	★	/	7
Ahlehoff et al. (4)	★	★	★	★	★★	★	★	/	8
Ahlehoff et al. (43)	★	★	★	★	★★	★	★	/	8
Ahlehoff et al. (42)	★	★	★	★	★★	★	★	/	8
Ahlehoff et al. (41)	★	★	★	★	★★	★	★	/	8
Brauchli et al. (37)	★	★	★	★	★★	★	★	/	8
Chiang et al. (21)	★	★	★	★	★★	★	★	/	8
Chiu et al. (22)	★	★	★	/	★★	★	★	★	8
Chiu et al. (23)	★	★	★	★	★★	★	★	/	8
Chung et al. (24)	★	★	★	★	★★	★	★	/	8
Dregan et al. (44)	★	★	★	★	★★	★	★	★	9
Egeberg et al. (45)	★	★	★	★	★★	★	★	/	8
Egeberg et al. (25)	★	★	★	★	★★	★	★	/	8
Gelfand et al. (39)	★	★	★	★	★★	★	★	/	8
Gelfand et al. (38)	★	★	★	★	★★	★	★	/	8
Jung et al. (26)	★	★	★	/	★★	★	★	★	8
Kaye et al. (40)	★	★	★	★	★★	★	★	/	8
Khalid et al. (27)	★	★	★	★	★★	★	★	/	8
Khalid et al. (46)	★	★	★	★	★★	★	★	/	8
Khalid et al. (47)	★	★	★	★	★★	★	★	/	8
Leisner et al. (28)	★	★	★	★	★	★	★	★	8
Levesque et al. (29)	★	★	★	/	★★	★	★	/	7
Lin et al. (30)	★	★	★	★	★★	★	★	/	7
Lin et al. (31)	★	★	★	★	★	★	★	/	7
Mehta et al. (12)	★	★	★	★	★★	★	★	★	9
Mehta et al. (32)	★	★	★	/	★★	★	★	★	8
Ogdie et al. (33)	★	★	★	★	★★	★	★	/	8
Parisi et al. (34)	★	★	★	/	★★	★	★	★	8
Rhee et al. (35)	★	★	★	★	★★	★	★	/	8
Wakkee et al. (36)	★	★	★	★	★★	★	★	/	8
Wu et al. (13)	★	★	★	★	★★	★	★	/	8

### Publication Bias

**FIGURE 2:**
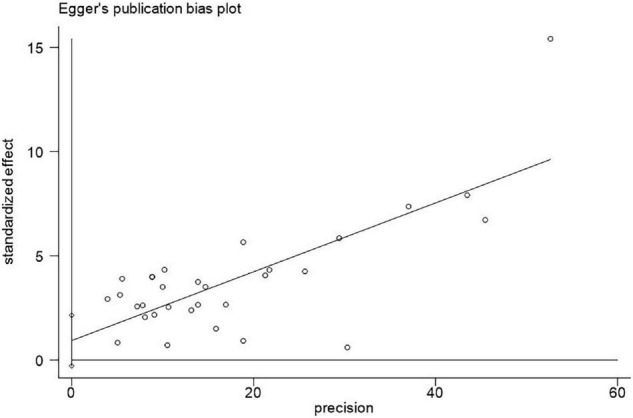
Egger’s test for publication bias of 31 included articles for adverse cardiovascular outcomes (*P* = 0.125).

We visually examined the funnel plot of the 31 articles and concluded that it was symmetrical (presented in Figure 2). Begg’s test showed a significant publication bias among these studies (*P* = 0.003). However, Egger’s test revealed that there was no obvious publication bias (*P* = 0.125). The Egger’s test was used in the present study since it is usually considered more sensitive than the Begg’s test (17).

### Pooled Analysis

**FIGURE 3:**
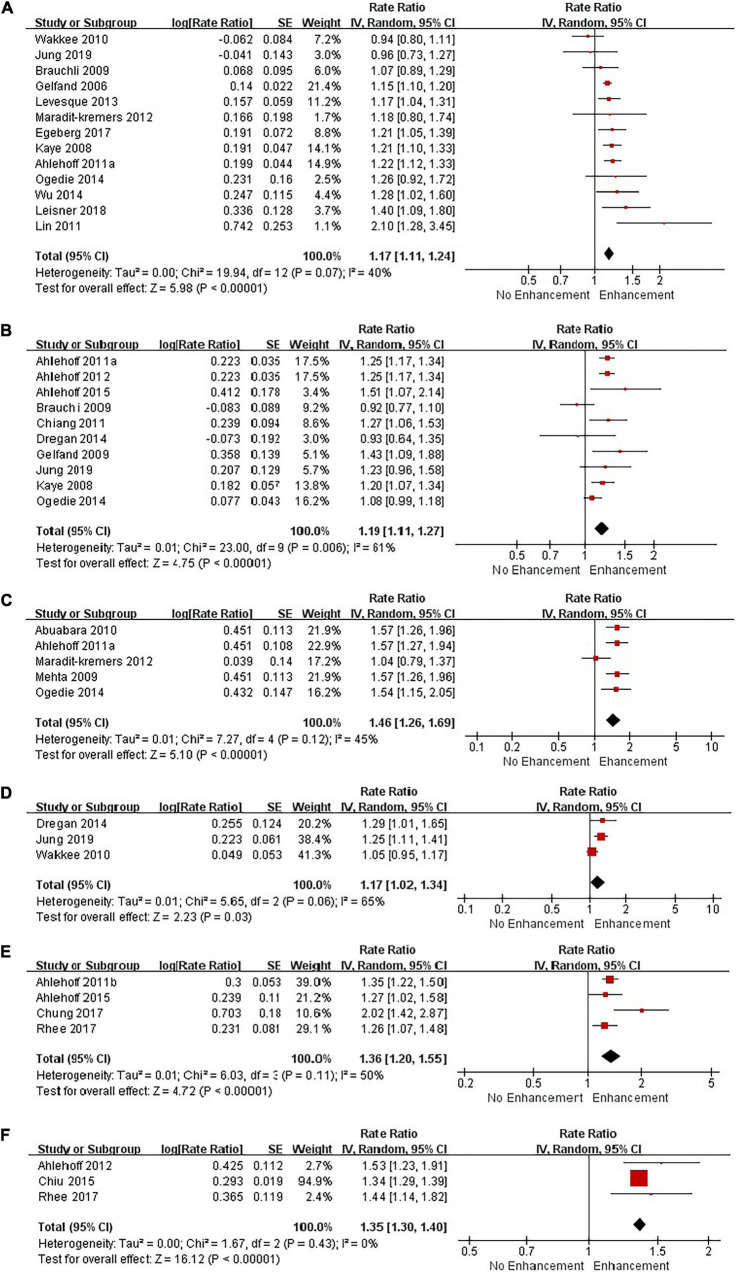
Forest plot of adverse cardiovascular outcomes based on Review Manager. **(A)** Risk of myocardial infarction in patients with psoriasis; **(B)** risk of stroke in patients with psoriasis; **(C)** risk of cardiovascular death in patients with psoriasis; **(D)** risk of ischemic heart disease in patients with psoriasis; **(E)** risk of thromboembolism in patients with psoriasis; **(F)** risk of arrhythmia in patients with psoriasis.

**FIGURE 4:**
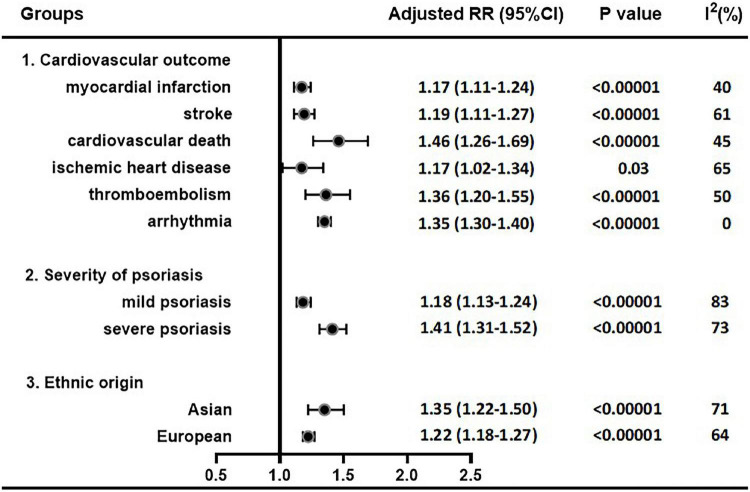
Pooled adjusted RR estimates in each cardiovascular outcome, psoriasis severity and ethnic origin.

We performed combined analysis in terms of each cardiovascular outcome, ethnic origin, and severity of psoriasis. Table 3 presents a summary of RR estimates, corresponding 95% CI, *Q*-test, and *I*^2^ estimates for each analysis (presented in Figures 3, 4).

**TABLE 3:** Estimates of RRs and the 95% CIs for adverse cardiovascular disease and heterogeneity test for studies with Cochran’s *Q*-test and the quantity *I*^2^.

Type	No. of data sets	No. of cases	No. of controls	RR	95% CI	*Q*-value	I^2^(%)	*P*-value
**Cadiovascular outcome**								
Myocardial infarction	13	522,730	11,171,909	1.17***	1.11–1.24	19.94	40	< 0.00001
Stroke	10	484,839	11,529,624	1.19***	1.11–1.27	23.00	61	< 0.00001
Cardiovascular death	5	183,966	4116,176	1.46***	1.26–1.69	7.27	45	< 0.00001
Ischemic heart disease	3	67,048	2,129,260	1.17*	1.02–1.34	5.65	65	0.03
Thromboembolism	4	63,236	4,971,594	1.36***	1.20–1.55	6.03	50	< 0.00001
Arrhythmia	3	93,580	5,380,933	1.35***	1.30–1.40	1.67	0	< 0.00001
Others	8	330,342	17,942,545	/	/	/	/	/
**Ethnic origin**								
Asian	7	110,591	2,813,658	1.35***	1.22–1.50	20.98	71	< 0.00001
European	24	554,418	15,089,099	1.22***	1.18–1.27	64.75	64	< 0.00001
**Severity of psoriasis**								
Mild	18	857,939	39,811,954	1.18***	1.13–1.24	100.75	83	< 0.00001
Moderate and severe	22	917,271	400,631,311	1.41***	1.31–1.52	78.75	73	< 0.00001

*P < 0.05, ***P < 0.0001.

#### Pooled Analysis of Main Cardiovascular Outcomes

##### Myocardial Infarction

Thirteen studies investigated the risk of myocardial infarction in patients with psoriasis, including 522,730 patients and 11,171,909 non-psoriatic control subjects. Our meta-analysis with random-effects model showed a moderately increased risk of myocardial infarction in patients with psoriasis (1.17; 95% CI 1.11–1.24). Heterogeneity test showed no significant difference among these studies (*Q* = 19.94 with 12 df, *P* = 0.07; *I*^2^ = 40%). Therefore, our meta-analysis showed that psoriasis was a related risk factor for myocardial infarction.

##### Stroke

Ten studies investigated the risk of stroke in patients with psoriasis, including 484,839 psoriasis patients and 11,529,624 non-psoriatic control subjects. Our meta-analysis showed a significant association of psoriasis with stroke (1.19; 95% CI 1.11–1.27). Heterogeneity test showed a difference among these studies (*Q* = 23.0 with 9 df, *P* = 0.006; *I*^2^ = 61%). Sensitivity analysis showed that statistical heterogeneity can be effectively removed (*Q* = 13.87 with 8 df, *P* = 0.09; *I*^2^ = 42%) when the data sets of a study (37) were excluded, and the result was consistent with the previous trend. Our meta-analysis showed that psoriasis was associated with an increased risk of stroke.

##### Cardiovascular Death

Five studies investigated the risk of cardiovascular death in patients with psoriasis in Europeans, including 183,966 psoriasis patients and 4,116,176 non-psoriatic control subjects. Heterogeneity test demonstrated that there was no heterogeneity among these studies (*Q* = 7.27 with 4 df, *P* = 0.12; *I*^2^ = 45%). Our meta-analysis with random-effects model showed a significantly increased risk of cardiovascular death in patients with psoriasis (1.46; 95% CI 1.26–1.69). Thus, our meta-analysis result showed that psoriasis was associated with an increased risk of cardiovascular death.

##### Ischemic Heart Disease

Three studies investigated the risk of ischemic heart disease in patients with psoriasis in Europeans, including 67,048 psoriasis patients and 2,129,260 non-psoriatic control subjects. The pooled RR estimate was 1.17 (95% CI 1.02–1.34). There was no significant heterogeneity when these data sets were analyzed together (*Q* = 5.65 with 2 df, *P* = 0.06; *I*^2^ = 65%), which showed psoriasis patients had a higher risk of ischemic heart disease.

##### Thromboembolism

Four studies investigated the risk of thromboembolism in patients with psoriasis, including 63,236 psoriasis patients and 4,971,594 non-psoriatic control subjects. The pooled RR estimate was 1.36 (95% CI 1.20–1.55). Heterogeneity tests showed no difference among these studies (*Q* = 6.03 with 3 df, *P* = 0.11; *I*^2^ = 50%). Our meta-analysis result showed that patients with psoriasis had a higher risk of thromboembolism.

##### Arrhythmia

Three studies investigated the risk of arrhythmia in patients with psoriasis, including 93,580 psoriasis patients and 5,380,933 non-psoriatic control subjects. The pooled RR estimate was 1.35 (95% CI 1.30–1.40). There was no significant heterogeneity when these data sets were analyzed together (*Q* = 1.67 with 2 df, *P* = 0.43; *I*^2^ = 0%), which showed psoriasis patients had a higher risk of arrhythmia.

##### Others

Eight studies involving 330,342 psoriasis patients and 17,942,545 non-psoriatic control subjects had addressed the risk of other adverse cardiovascular outcomes in psoriasis patients, including heart failure, angina, aortic aneurysm, aortic valve stenosis, atherosclerosis and transient ischemic attack. The pooled analysis of these outcomes was not performed since the studies were insufficient and the results were poorly represented.

#### Pooled Analysis in Terms of Ethnic Origin

Seven studies investigated the risk of CVD or CVE in Asian patients with psoriasis, including 110,591 psoriasis patients and 2,813,658 non-psoriatic control subjects; twenty-four studies in European patients, including 554,418 psoriasis patients and 15,089,099 non-psoriatic subjects. We found increased adverse cardiovascular outcomes risks in European (1.22; 95% CI, 1.18–1.27) and Asian (1.35; 95% CI, 1.22–1.50) groups, but there was no significant difference between two groups (*P* = 0.076). After eliminating heterogeneity, the combined RR estimates for the Asian and European groups were 1.33 (95% CI, 1.29–1.38) and 1.24 (95% CI, 1.20–1.28), respectively.

#### Pooled Analyses in Terms of Severity of Psoriasis

Eighteen studies detected the risk of CVD or CVE in patients with mild psoriasis, involving 857,939 psoriasis patients and 39,811,954 non-psoriatic subjects; 22 studies involving 917,271 psoriasis patients and 40,063,131 non-psoriatic subjects detected in moderate and severe psoriasis patients. Our results revealed a significantly increased risk in both mild (1.18; 95% CI 1.13–1.24) and moderate-to-severe patients (1.41; 95% CI 1.31–1.52), with a higher RR in moderate-to-severe patients (*P* = 0.001). The result of heterogeneity test showed significant differences among these studies. After eliminating heterogeneity, the combined RR estimates were 1.20 (95% CI, 1.16–1.24) for mild psoriasis and 1.41 (95% CI, 1.33–1.50) for moderate and severe psoriasis.

### Certainty of Evidence Assessment

Evidence assessment was performed using GRADE profiler, and grades of cohort studies were all slightly lower than randomized controlled trials (RCTs). However, we believe cohort studies provided the highest level of evidence for the meta-analysis conducted in the present study based on the following considerations. (1) The present systematic review included 31 high-quality cohort studies according to the NOS scale, and all low-quality studies had been excluded. (2) RCT was not applicable to the etiological research of CVDs due to ethical constraints.

## Discussion

This research was a large-scale cohort meta-analysis of the correlation between psoriasis and CVD that included 31 cohort studies involving 665,009 psoriasis patients and 17,902,757 non-psoriatic control subjects. Our meta-analysis revealed that psoriasis patients had significantly increased the risk for developing adverse cardiovascular outcomes. Patients with psoriasis, especially severe psoriasis, had a higher risk of ischemic heart disease, myocardial infarction, stroke, thromboembolism, arrhythmia and cardiovascular death.

Samarasekera et al. conducted a meta-analysis (15) included 14 cohort studies, 7 of them met our inclusion criteria and were included in our meta-analysis. The results of their analysis indicated that the risk of CVD was significantly related to severe psoriasis (HR, 1.57; 95% CI, 1.26–1.96), which was consistent with our findings. We found that cardiovascular morbidity was also significantly increased in patients with mild psoriasis compared with non-psoriatic subjects, which was not observed in their study. Another published meta-analysis (14) that included 75 observational studies (38 cross-sectional studies, 32 case–control studies, and 5 cohort studies) arrived at a similar conclusion with us only in case–control studies.

According to the World Health Organization, CVD is a group of disorders of the heart and blood vessels, including coronary artery disease (angina pectoris, myocardial infarction, etc.), peripheral arterial disease (stroke), rheumatic heart disease, and congenital heart disease. Traditional risk factors for CVD include hypertension, dyslipidemia, smoking, obesity, diabetes etc. (56). To recognize the role of psoriasis for adverse cardiovascular outcomes, it was necessary to adjust for the influence of as many known traditional factors as possible. In our study, 27 of the included studies had adjusted CVD traditional factors, although the type and number of factors varied from study to study, which included age, gender, socioeconomic status, prior CVD, current smoking, body mass index, total cholesterol etc. Thus, our conclusions had a high degree of credibility.

At present, there are a number of clinical options for the treatment of psoriasis, including TNF-α inhibitors, oral/phototherapy, systematic treatment with traditional medicine, and topical treatments (57–61). Different treatment modalities may affect the cardiovascular risk of patients with psoriasis. A study demonstrated that treatment of psoriasis with TNF-α inhibitors (etanercept, infliximab, or adalimumab) significantly reduced the risk and incidence of myocardial infarction compared with treatment with topical medication. Also, treatment of psoriasis with TNF inhibitors had no significant difference in the incidence of myocardial infarction compared with treatment with oral medications (i.e., cyclosporine, acitretin, and methotrexate)/phototherapy (59). In a national study of patients with severe psoriasis (57), it was shown that systemic anti-inflammatory therapy with biologics (TNF-α inhibitors and interleukin inhibitors) or methotrexate significantly reduced the incidence of CVD compared with patients treated with other anti-psoriatic therapies. Interestingly, two published meta-analyses of 22 and 38 RCTs concluded that there was no significant difference in the incidence of major adverse CVE between biologically treated patients and placebo patients (62, 63). Due to the lack of optimal random-effects modeling methods, the poor data quality, and the short control periods of the experiment, their conclusions had subsequently been questioned, and even the authors themselves share this concern (61). Therefore, different treatment modalities are likely to alter the cardiovascular risk of patients with psoriasis. The lack of data on the treatment of patients with psoriasis in our included studies may be a reason for the heterogeneity.

This research also has several limitations. Above all, the association between psoriasis and ischemic heart disease may be greatly underestimated. As for ischemic heart disease, all three studies included in our meta-analysis used ICD to identify the disease, but in silent ischemic cases, it could only be detected by coronary angiography (CAG) or CT angiography (CTA). Given that it’s unknown whether all subjects received CAG or CTA on a regular basis, some cases of ischemic heart disease may remain undetected, resulting in significant underestimation of the true relationship between psoriasis and ischemic heart disease. Furthermore, our results indicated that the strongest association occurred in the cardiovascular mortality group. However, we found that four of the five included studies were of severe psoriasis patients, which may have led to an overestimation of the risk of cardiovascular mortality from psoriasis. These limitations may be sources for heterogeneity.

Nonetheless, our meta-analysis holds some key strengths in several aspects. Firstly, due to ethical constraints, cohort studies are the most appropriate research method for comorbidity studies. Therefore, the highest level of evidence was provided in the present systematic review of cohort studies. Secondly, large numbers of psoriasis patients and non-psoriatic subjects were pooled from 31 included studies, which significantly improved the statistical power of the analysis. Thirdly, according to the NOS scale, the quality of cohort studies included in the present study was high (≥ 7 points). Fourthly, we performed a more comprehensive analysis and further confirmed that psoriasis was associated with multiple adverse cardiovascular outcomes.

We recommend that clinicians, while treating patients with psoriasis, pay more attention to the cardiovascular risk of patients. First, we call on doctors or communities to educate people at a high risk for CVD on prevention so people can care for their health. In a way, the guidelines for cardiovascular prevention may also be valid for psoriasis patients. This is an enhanced primary prevention of CVD, which is a guideline to reduce the incidence of CVD in patients with psoriasis. An RCT of 303 cases revealed that a 20-week dietetic intervention associated with increased physical exercise reduced psoriasis severity in systemically treated overweight or obese patients with active psoriasis (64). Meanwhile, several studies had found that exercise not only reduces the risk of psoriasis, but also promotes cardiovascular health (65, 66). Exercise avoidance in patients with psoriasis can lead to an increased risk of CVD (67). Second, regular stress-testing to monitor the presence of coronary artery disease may be a useful screening for psoriasis patients.

## Conclusion

Overall, our meta-analysis found that psoriasis was associated with an increased risk of all pooled observed adverse cardiovascular outcomes regardless of ethnic origin or severity, including myocardial infarction, stroke, thromboembolism, arrhythmia and cardiovascular death. This association was even stronger in moderate-to-severe psoriasis. This suggested that psoriasis may be an independent risk factor for all adverse CVE, which requires more attention from clinicians.

## Data Availability Statement

The original contributions presented in the study are included in the article/Supplementary Material, further inquiries can be directed to the corresponding author/s.

## Author Contributions

GZ and NW: supervision, concept, and design. GZ, NW, XH, and LL: acquisition, analysis, or interpretation of data. LL and SC: drafting of the manuscript. LL and ML: statistical analysis. All authors critical revision of the manuscript for important intellectual content and have approved the final manuscript.

## Conflict of Interest

The authors declare that the research was conducted in the absence of any commercial or financial relationships that could be construed as a potential conflict of interest.

## Publisher’s Note

All claims expressed in this article are solely those of the authors and do not necessarily represent those of their affiliated organizations, or those of the publisher, the editors and the reviewers. Any product that may be evaluated in this article, or claim that may be made by its manufacturer, is not guaranteed or endorsed by the publisher.

## Footnotes

1
http://www.ohri.ca/programs/clinical_epidemiology/oxford.asp
