# Corrigendum: Psoriasis Increased the Risk of Adverse Cardiovascular Outcomes: A New Systematic Review and Meta-Analysis of Cohort Study

**DOI:** 10.3389/fcvm.2022.929149

**Published:** 2022-05-13

**Authors:** Lu Liu, Saijin Cui, Meitong Liu, Xiangran Huo, Guoqiang Zhang, Na Wang

**Affiliations:** ^1^Cancer Institute, The Fourth Hospital of Hebei Medical University, Shijiazhuang, China; ^2^Department of Dermatology, The First Hospital of Hebei Medical University, Shijiazhuang, China; ^3^Candidate Branch of National Clinical Research Center for Skin Diseases, Beijing, China

**Keywords:** cardiovascular disease, cohort study, meta-analysis, systematic review, psoriasis

In the original article, the e-mails of two communication authors were in the wrong order.

The authors apologize for this error and state that this does not change the scientific conclusions of the article in any way. The original article has been updated.

## Publisher's Note

All claims expressed in this article are solely those of the authors and do not necessarily represent those of their affiliated organizations, or those of the publisher, the editors and the reviewers. Any product that may be evaluated in this article, or claim that may be made by its manufacturer, is not guaranteed or endorsed by the publisher.

