# Supplementary material for: Psoriasis Increased the Risk of Adverse Cardiovascular Outcomes: A New Systematic Review and Meta-Analysis of Cohort Study
Source: Front Cardiovasc Med. 2022 Mar 25;9:829709. doi: 10.3389/fcvm.2022.829709 (PMC8990932; doi:10.3389/fcvm.2022.829709)
Supplement: Supplementary file 1 [file Data_Sheet_1.PDF]

Supplementary Table S1. Retrieval strategies used to filter literatures in each database.

**Database**	**Retrieval Strategies**	**Result**
1.MEDLINE	#1：("Psoriasis"[MeSH Terms] OR “Psoriases”[Title/Abstract] OR “Pustulosis Palmaris et Plantaris”[Title/Abstract] OR “Palmoplantaris Pustulosis”[Title/Abstract] OR “Pustular Psoriasis of Palms and Soles”[Title/Abstract])<br>#2：("cardiovascular diseases"[MeSH Terms] OR “Cardiovascular Disease”[Title/Abstract] OR “Disease, Cardiovascular”[Title/Abstract] OR “Diseases, Cardiovascular”[Title/Abstract])<br>#3：("risk"[Title/Abstract] OR "risk"[MeSH Terms:noexp] OR "mortality"[Title/Abstract] OR "mortality"[MeSH Terms:noexp] OR "cohort"[Title/Abstract])<br>#4：#1 AND #2 AND #3	1051
2. SCI-Web of Science	#1：("Psoriasis"[MeSH Terms] OR “Psoriases”[Title/Abstract] OR “Pustulosis Palmaris et Plantaris”[Title/Abstract] OR “Palmoplantaris Pustulosis”[Title/Abstract] OR “Pustular Psoriasis of Palms and Soles”[Title/Abstract])<br>#2：("cardiovascular diseases"[MeSH Terms] OR “Cardiovascular Disease”[Title/Abstract] OR “Disease, Cardiovascular”[Title/Abstract] OR “Diseases, Cardiovascular”[Title/Abstract])<br>#3：("risk"[Title/Abstract] OR "risk"[MeSH Terms:noexp] OR "mortality"[Title/Abstract] OR "mortality"[MeSH Terms:noexp] OR "cohort"[Title/Abstract])<br>#4：#1 AND #2 AND #3	896
3. EMBASE	#1：'cardiovascular disease':ti,ab,kw OR 'disease, cardiovascular':ti,ab,kw OR 'diseases, cardiovascular':ti,ab,kw<br>#2：'psoriasis'/exp<br>#3：psoriasis:ab,ti OR psoriases:ab,ti OR (pustulosis of palms:ab,ti AND soles:ab,ti) OR pustulosis palmaris et plantaris:ab,ti OR palmoplantaris pustulosis:ab,ti OR (pustular psoriasis of palms:ab,ti AND soles:ab,ti)<br>#4：'risk'/de OR 'mortality'/de OR 'cohort':ti,ab<br>#5：#1 AND #2 AND #3 AND #4	590
4.The Cochrane Library	#1：MeSH descriptor: [Cardiovascular Diseases] explode all trees<br>#2：MeSH descriptor: [Psoriasis] explode all trees<br>#3：("risk" OR mortality OR cohort):ti,ab,kw<br>#4: #1 AND #2 AND #3	27
